# Disease severity-based evaluation of utility weights for lung cancer-related health states in Korea

**DOI:** 10.1186/s12885-018-4960-y

**Published:** 2018-11-08

**Authors:** Eun-Jung Kim, Minsu Ock, Kyu-pyo Kim, Nuri Hyun Jung, Hyeon-Jeong Lee, Seon-Ha Kim, Min-Woo Jo

**Affiliations:** 1 Department of Nursing, Pyeongtaek University, Pyeongtaek, Gyeonggi South Korea; 2 Department of Preventive Medicine, Ulsan University Hospital, University of Ulsan College of Medicine, Ulsan, South Korea; 3 Department of Internal Medicine, Asan Medical Center, University of Ulsan College of Medicine, Seoul, South Korea; 4 Department of Radiation Oncology, Asan Medical Center, University of Ulsan College of Medicine, Seoul, South Korea; 5 Department of Preventive Medicine, University of Ulsan College of Medicine, 88 Olympic-ro 43-gil, Songpa-Gu, Seoul, 05505 South Korea; 6 Department of Nursing, College of Nursing, Dankook University, Cheonan, South Korea

**Keywords:** Utility, Health-related quality of life, Lung cancer, Squamous cell carcinoma

## Abstract

**Background:**

Utility weight, a measure of health-related quality of life, is used in disease burden measurements and economic evaluations. In this study, we used the visual analogue scale (VAS) and standard gamble (SG) method to determine the utility weights of lung cancer health states in South Korea from a societal perspective.

**Methods:**

Six hypothetical health states for lung cancer or a related health state reflective of disease severity were developed: 1) Stage I, 2) Stage II, 3) Stage IIIa, 4) Stage IIIB, 5) Stage IV, and 6) Pulmonary nodule. The description of each health state description was divided into four parts: diagnosis, symptoms, treatment, and progression and prognosis. A total of 515 representative adult Korean participants used a VAS and SG to evaluate these six health states via face-to-face computer-assisted interviews. The means, standard deviations, and median utility weights of the six health states were estimated by valuation method.

**Results:**

The two valuation methods of the scenarios yielded the same mean utility rankings. Pulmonary nodule received the highest rank (VAS, 0.66 and SG, 0.83), whereas Stage 4 was assigned the lowest rank (VAS, 0.09 and SG, 0.31). For all health states, the mean utility weights calculated using the SG were greater than those calculated using the VAS. The differences between the utility weights obtained using the two valuation methods ranged from 0.14 (Stage I) to 0.22 (Stage IV). The two approaches tended to yield larger differences for more severe stages.

**Conclusions:**

This study determined utilities for squamous cell lung cancer that will be useful for estimating the burden of lung cancer and for conducting economic evaluations of lung cancer interventions.

**Electronic supplementary material:**

The online version of this article (10.1186/s12885-018-4960-y) contains supplementary material, which is available to authorized users.

## Background

In South Korea, 8.44% of the total disability-adjusted life years have been attributed to cancer. Currently, lung cancer is the most frequent malignancy, accounting for 14.05% of all cancer cases [1]. Although lung cancers are associated with symptoms such as cough, dyspnea, weight loss, and chest tightness, patients with very early-stage disease may be asymptomatic. In short, disease progression correlates inversely with the quality of life [2]. Furthermore, lung cancer has a higher mortality rate, compared to other diseases; accordingly, the years of life lost (YLL) is higher than the years lost to disability (YLD), and the YLL:YLD ratio is asymmetric [3].

Utility weight, an indicator of the health-related quality of life, is used in measurements of disease burdens and economic evaluations, and the results provide important evidence to support the decision-making processes of policy makers [4, 5]. However, the quantification of utility weight is a somewhat vague and non-objective process because of the target population [6]. Ideally, decision makers should have access to utility data specific to local populations [7]. A preference-based assessment, which can be categorized into direct and indirect methods, can be used to achieve this ideal [8].

Several methods can be used to elicit a health state preference. In the present study, we used the visual analogue scale (VAS) and standard gamble (SG) techniques. SG methods require concentration and in-depth cognitive functions from the respondents and professional interviewers [9, 10]. These methods use self-administered, multi-attribute health status classification system questionnaires and have been widely used [8]. The alternative method, VAS, is simple. However, the VAS anchors are often poorly defined, and may lead to several measurement biases, such as context and end-aversion bias. However, the VAS is a value but utility and thus might be used to compare preferences and explore methodological validity. Evidence suggests that limited and cautious use of the VAS is useful and appropriate [11].

This study, therefore, aimed to determine the utility weights of squamous cell lung cancer health states in South Korea from a societal perspective. These utility weights were determined to provide local population data to policy makers in South Korea, as well as internationally.

## Methods

### Health states

Two authors (Ock M and Jo MW) developed a draft of the lung cancer health state scenarios based on literature reviews and the education materials provided to patients at Asan Medical Center. One medical oncologist (Kim KP) and one radiation oncologist (Jung NH) reviewed and modified the draft. A total of six hypothetical health states reflecting disease severity were developed: 1) Stage I lung cancer (state 1), 2) Stage II lung cancer (state 2), 3) Stage IIIa lung cancer (state 3), 4) Stage IIIb lung cancer (state 4), 5) Stage IV lung cancer (state 5), and 6) Pulmonary nodule (state 6). The histological type of lung cancer was assumed to be squamous cell carcinoma. Each health state was divided into four parts with reference to previous studies [12–14]: diagnosis, symptoms, treatment, and progression and prognosis. Specifically, each health state included standard diagnostic procedures, typical symptoms and signs, standard treatment procedures (including surgery and radiotherapy), common side effects of treatment, and information about the 5-year survival rate. The full descriptions of the six health states are available in Additional File 1.

### Study participants

The target population comprised adults aged ≥19 years who lived in Korea (except Jeju Island). This target population was defined in the June 2013 national resident registration data, documented by the Ministry of Administration and Security of South Korea. We recruited 515 representative individuals from this target population using a multi-stage stratified quota sampling based on region, sex, and age group.

### Survey procedure

Participants were surveyed through face-to-face computer-assisted interviews. Before conducting the survey, the interviewers were educated about the purpose of the study and the descriptions of the six health states. They were also trained in two valuation methods: VAS and SG. The total education time was approximately 2.5 h. The sample script of the interview was available in Additional File 2.

Every participant was informed about the survey procedure and the purpose of the study, and those who provided informed consent were included in this survey. First, participants were requested to provide their age, sex, region, and educational level. Next, participants applied the VAS six times to the six health states, which were displayed in a random order. The participants also applied the VAS once more for the state of death. Next, the participants used the SG method to evaluate the six health states in random order. After performing the two valuations, the participants were asked about other socio-demographic factors and clinical information, including their monthly income, ambulatory care visits during the past 2 weeks, hospitalization during the past 12 months, and morbidity. The health state descriptions were printed on cards to facilitate participants with limited vision. The Institutional Review Board (IRB) of Asan Medical Center determined that this study was exempt from IRB review (approval number: S2016–0015).

### Valuation methods

Two valuation methods were used in this study: VAS and SG. For the VAS, the participants rated the proposed health states on a scale from 0 to 100, which corresponded to the worst and best imaginable health states, respectively. For the SG, the participants were asked to select the better of two choices: either a health state or death. If the participant chose the proposed health state as the better option, he/she was asked to determine the point of indifference between two hypothetical options: remaining in the proposed health state for the remaining life time, or receiving treatment that might lead to either a full recovery of health (probability ‘p’) or immediate death (probability ‘1-p’). The probability ‘p’ increased or decreased by 5% according to the previous participants’ choice. The participants continued to select options until their preferences for the options became equal. The initial probability ‘p’ was 50%. If the participant selected death as a preferred state, the next SG question was presented. VAS and SG were different from EQ-5D, HUI and SF-6D. The later ones were indirect evaluation methods so it could be interpreted multiply. Especially, in the field of clinical setting, the direct evaluation methods were more preferred [15].

### Data analysis

We performed descriptive analyses of socio-demographic factors and clinical information. Utility weights were determined using each valuation method. For the VAS, utility weights were calculated using the following formula: (VAS value of the health state – VAS value of death) / (100 – VAS value of death). For the SG, if the health state was selected as better than death, the final value of ‘p’ was recorded as the utility weight of the suggested health state. If the health state was selected as a worse than death, the utility was censored at 0.

The mean, standard deviation, and median utility weights of six health states were estimated by valuation method. We used Student’s t-test and an analysis of variance to compare the mean values of utilities according to the socio-demographic factors and clinical information. Furthermore, we used a linear mixed model to identify factors that influenced the utility weights. The utility weights obtained using the VAS and SG methods were regarded as dependent variables, and the socio-demographic factors, clinical information, and health states were treated as independent variables. All statistical analyses were performed using SPSS software (v21.0; SPSS, Inc., Chicago, IL, USA) and SAS software (v9.2; SAS Institute, Inc., Cary, NC, USA). *P*-values < 0.05 were considered statistically significant.

## Results

A total of 515 participants completed full interviews. The mean age of the participants was 45.7 (SD: 13.9) years, and 48.9% were male. Among the participants, 13.2% have received ambulatory cares during the past 2 weeks, and 3.1% had been hospitalized within the previous 12 months. The clinical and demographic characteristics of the participants are shown in Table 1.

**Table 1:** Characteristics of the participants (*N* = 515)

Characteristics		N	%
Sex	Male	252	48.9
	Female	263	51.1
Age group (years)	19–29	90	17.5
	30–39	92	17.9
	40–49	112	21.7
	50–59	105	20.4
	≥60	116	22.5
Education level	Middle school or below	40	7.8
	High school	245	47.0
	College or above	230	44.7
Occupation	Non-manual	124	24.1
	Manual	263	51.1
	Other	128	24.9
Monthly income (Korean won)	< 2.5 million	88	17.1
	2.5–5 million	337	65.4
	> 5.0 million	90	17.5
Ambulatory care visit in past 2 weeks	Yes	68	13.2
	No	447	86.8
Hospitalization in past 12 months	Yes	16	3.1
	No	499	96.9
Morbidity	Yes	59	11.5
	No	456	88.5

The utility weights for the lung cancer scenarios are shown in Table 2. The ranks of the scenarios by mean utility were the same, regardless of the valuation method. Pulmonary nodule was assigned the highest rank, with a VAS value of 0.66 and SG value of 0.83, whereas Stage IV was assigned the lowest rank, with a VAS value of 0.09 and SG value of 0.31. For all health states, the mean utility calculated using the SG method was greater than that calculated using the VAS. The differences in utility weights between the two valuation methods ranged from 0.14 (Stage I) to 0.22 (Stage IV). Severe stages tended to be associated with greater differences between the two approaches.

**Table 2:** Utility weights of lung cancer states derived using the visual analogue scale and standard gamble

States	Visual analogue scale			Standard gamble		
	Mean	Standard deviation	Median	Mean	Standard deviation	Median
State 1: Stage I	0.48	0.17	0.50	0.66	0.27	0.70
State 2: Stage II	0.38	0.17	0.40	0.56	0.28	0.60
State 3: Stage IIIa	0.27	0.17	0.28	0.45	0.29	0.40
State 4: Stage IIIB	0.20	0.18	0.20	0.38	0.29	0.30
State 5: Stage IV	0.09	0.18	0.10	0.31	0.30	0.20
State 6: Pulmonary nodule	0.66	0.21	0.70	0.83	0.24	0.90

Tables 3 and 4 presents a comparison of the utility weights according to socio-demographic and clinical information. The mean utility weights derived from the SG method did not differ significantly according to sex, age, educational level, occupation, and ambulatory care visits during the past 2 weeks. However, participants with higher monthly incomes tended to score higher than those with lower incomes. In addition, significantly higher rates for Pulmonary nodule were observed among participants with no hospitalizations during the previous 12 months and those without comorbidities.

**Table 3:** Utility weights from VAS according to socio-demographic factors (N = 515)

		State 1	State 2	State 3	State 4	State 5	State 6
Sex	Male	0.48	0.38	0.27	0.20	0.09	0.66
	Female	0.48	0.38	0.27	0.20	0.10	0.66
Age group (years)	19–29	0.47	0.37	0.26	0.18	0.08	0.64
	30–39	0.49	0.39	0.31	0.23	0.11	0.66
	40–49	0.49	0.37	0.26	0.19	0.08	0.67
	50–59	0.48	0.37	0.26	0.20	0.10	0.66
	≥60	0.48	0.38	0.26	0.19	0.09	0.67
Education level	High school or below	0.48	0.38	0.27	0.20	0.09	0.67
	College or above	0.48	0.37	0.27	0.19	0.09	0.65
Occupation	Non-manual	0.46	0.34	0.25	0.18	0.07	0.63
	Manual	0.49	0.38	0.28	0.21	0.10	0.66
	Other	0.49	0.39	0.27	0.19	0.10	0.69
Monthly income (Korean won)	< 2.5 million	0.48	0.38	0.23	0.17	0.07	0.66
	2.5–5.0 million	0.48	0.37	0.28	0.20	0.10	0.67
	> 5.0 million	0.48	0.38	0.27	0.22	0.10	0.63
Ambulatory care visit in past 2 weeks	Yes	0.50	0.39	0.27	0.20	0.08	0.68
	No	0.48	0.37	0.27	0.20	0.09	0.66
Hospitalization in past 12 months	Yes	0.48	0.36	0.25	0.19	0.05	0.61
	No	0.48	0.38	0.27	0.20	0.09	0.66
Morbidity	Yes	0.49	0.38	0.26	0.18	0.08	0.66
	No	0.48	0.38	0.27	0.20	0.09	0.66
VAS, visual analogue scale

**Table 4:** Utility weights from SG according to socio-demographic factors (N = 515)

		State 1	State 2	State 3	State 4	State 5	State 6
Sex	Male	0.66	0.55	0.45	0.38	0.31	0.82
	Female	0.65	0.57	0.46	0.37	0.31	0.83
Age group (years)	19–29	0.66	0.55	0.50	0.36	0.31	0.83
	30–39	0.62	0.55	0.44	0.37	0.30	0.83
	40–49	0.67	0.57	0.44	0.36	0.29	0.82
	50–59	0.66	0.54	0.44	0.39	0.33	0.82
	≥60	0.66	0.57	0.46	0.40	0.31	0.85
Education level	High school or below	0.66	0.56	0.45	0.38	0.31	0.83
	College or above	0.65	0.56	0.46	0.37	0.30	0.83
Occupation	Non-manual	0.67	0.57	0.48	0.40	0.35	0.82
	Manual	0.66	0.55	0.44	0.37	0.29	0.83
	Other	0.65	0.56	0.47	0.37	0.30	0.83
Monthly income (Korean won)	< 2.5 million	0.69	0.59	0.47	0.36 ^*^	0.27	0.84
	2.5–5.0 million	0.64	0.54	0.44	0.36 ^*^	0.31	0.81
	> 5.0 million	0.68	0.60	0.48	0.45 ^*^	0.35	0.88
Ambulatory care visit in past 2 weeks	Yes	0.71	0.61	0.47	0.42	0.28	0.87
	No	0.65	0.55	0.45	0.37	0.31	0.82
Hospitalization in past 12 months	Yes	0.59	0.24	0.33	0.27	0.21	0.75*
	No	0.66	0.28	0.46	0.38	0.31	0.83*
Morbidity	Yes	0.61	0.53	0.40	0.38	0.25	0.79*
	No	0.66	0.56	0.46	0.38	0.32	0.83*

*SG* standard gamble

^*^ P-value < 0.05

Table 5 presents the associations of various factors with the utility weights obtained using the VAS and SG methods. In this table, we have listed the results from a multivariable linear regression analysis. The health states were the main relevant factors related to the utility weights. After adjusting for clinical and demographic characteristics, the utility weight for a more advanced stage lung cancer relative to a pulmonary nodule was 0.568 and 0.519 lower when the VAS and SG methods were used, respectively. Furthermore, disease stage correlated inversely with utility weight. In addition, participants with manual occupations had a significantly higher utility weight when compared to those with a non-manual occupation only when the VAS was used.

**Table 5:** Linear mixed model of factors influencing utility weight

Factors	Visual analogue scale			Standard gamble		
	Coefficient	95% CI		Coefficient	95% CI	
Sex						
Female	−0.006	− 0.034	0.022	0.005	−0.034	0.044
Age (years)						
30–39	0.036	−0.011	0.082	−0.022	−0.087	0.042
40–49	0.011	−0.034	0.056	−0.018	−0.080	0.045
50–59	0.007	−0.039	0.054	−0.002	−0.066	0.063
≥ 60	0.001	−0.049	0.051	0.009	−0.061	0.079
Occupation						
Manual	**0.036**	0.000	0.072	−0.027	−0.077	0.023
Other	**0.042**	0.000	0.084	−0.030	−0.088	0.028
Education level						
College and above	−0.003	−0.036	0.030	−0.005	− 0.051	0.042
Monthly income (Korean won)						
2.5–5.0 million	0.010	−0.033	0.053	−0.028	−0.088	0.032
> 5.0 million	0.007	−0.041	0.054	−0.024	−0.089	0.042
Ambulatory care visit in past 2 weeks						
No	−0.014	−0.057	0.028	−0.054	− 0.114	0.005
Hospitalized in past 12 months						
No	0.025	−0.054	0.104	0.098	−0.011	0.208
Morbidity						
No	0.006	−0.040	0.053	0.064	−0.001	0.129
State						
State 1	**−0.177**	−0.190	− 0.164	**−0.172**	− 0.197	−0.148
State 2	**−0.284**	−0.297	− 0.271	**−0.269**	− 0.294	−0.245
State 3	**−0.392**	−0.405	− 0.379	**−0.374**	− 0.398	−0.349
State 4	**−0.462**	−0.475	− 0.449	**−0.451**	− 0.475	−0.426
State 5	**−0.568**	−0.581	− 0.555	**−0.519**	− 0.544	−0.495

*CI* confidence interval

Bold text indicates statistical significance

## Discussion

In this study, utility weights for six health states of lung cancer were determined using the VAS and SG methods by 515 participants from the general South Korean population. One strength of our study is the subject, namely the calculation of utility weight for lung cancer in an East Asian country. Few previous studies have evaluated the utility of lung cancer in general populations. However, some studies have recommended the use of utility weights from a general population [14, 16].

Nevertheless the strength, Froberg et al. found that differences in the measurement of social preference for health state vary among people involved in the measurement. The reason for this difference arises from the differences in knowledge or experience people have about the health state or disease. Therefore, in order to measure social value or preference properly, pre-training of health state or disease is required before. This can solve the problem that may arise from preconceived notions about the health state or disease that the public had in advance [17–19]. In this study, we developed the health descriptions for six health states of lung cancer, and explained them to the participants in the survey to improve understanding of health states.

Our study group used both VAS scale- and SG-based approaches to determine the utility weights for lung cancer-related health states. The SG approach measures respondents’ preferences under conditions of uncertainty and is based directly on the von Neumann–Morgenstern utility theory, which is considered the standard for modeling rational behavior in the context of uncertainty [20]. Although the VAS does not allow a trade-off between different health states, and is commonly considered to lack a theoretical basis when compared with choice-based methods such as SG and time trade-off (TTO) [21], it is easy to understand and use. Accordingly, the VAS is widely used in evaluation studies. Furthermore, no single method of evaluation is most appropriate under all circumstances [22]. However, the VAS tends to yield bias-prone utility weights and should not be used by itself [11]. Some studies have confirmed the context bias of this method [11] and identified the range frequency model developed by Parducci and Wedell as potentially useful for bias adjustment [23]. However, it is difficult to generalize this model [23]; therefore, to ensure the best outcome, the VAS should be used only as an introductory method during which respondents can become accustomed to the health states. The ordinal preferences can facilitate the subsequent SG analysis [11, 24].

We evaluated lung cancer-related health state preferences in a relatively large sample, using various scenarios and direct valuation methods. The lung cancer utility weights ranged from 0.09 (stage IV lung cancer) to 0.83 (pulmonary nodule); notably, more severe states received consistently lower values. A previous study [25] reported a utility weight of 0.626 for stable disease without additional symptoms and decreases of 0.069 with the addition of pain, 0.050 with dyspnea, and 0.046 with cough; the utility values ranged from 0.653 for stable disease with no toxicity to 0.473 for progressive disease [26].

Our utility weights are similar to those reported in a previous study [13]. A meta-regression analysis determined reference lung cancer utilities of 0.573, 0.772, and 0.823 for metastatic, mixed/not specified, and non-metastatic non-small cell lung cancer (NSCLC), respectively, using patients as respondents and the SG method with scale boundaries of death and perfect health [27]. Using the TTO, Swinburn et al. [28] elicited health state utility weights for the treatment outcomes of relapsed/refractory Hodgkin lymphoma and systemic anaplastic large-cell lymphoma in Thailand, Taiwan, and South Korea. The values assigned for stable disease were 0.30 (Thailand), 0.49 (Taiwan)m and 0.64 (South Korea), which were comparable to the stable disease values for Korea (0.71) and Taiwan (0.54) according to a study by Nafee et al. [7]. The utility weight for progressive disease approached that of “death” in Thailand (0.07), and was low in both Taiwan (0.23) and South Korea (0.32) [13]. The current values for progressive disease are in line with the range observed in our study.

Our study proved the association between utility values obtained with the VAS and SG. Health states were the most relevant factors regarding utility weight. After we adjusted for clinical and demographic characteristics, the utility weight for higher-stage lung cancer relative to pulmonary nodule was 0.568 lower with the VAS and 0.519 lower with the SG. In addition, respondents with manual occupations yielded significantly higher utility weights than did those with non-manual occupation only when using the VAS. In other words, the risk attitudes may differ among respondents in terms of lung cancer severity, compared to other conditions, and other patient characteristics might supersede the experience of a health state. Furthermore, other cancer patients also ranked social functioning as more important than physical functioning. The current study adapted the existing health state descriptions of metastatic breast cancer from a previous study [29] to describe patients receiving second-line treatment for NSCLC. The utility values were obtained by asking participants to value health state descriptions describing metastatic breast cancer, five grade III/IV toxicities (febrile neutropenia, stomatitis; diarrhea and vomiting; fatigue; hand-foot syndrome), and hair loss [29]. The utility scores reflect the values assigned by ordinary people to various health states representative of lung cancer scenarios.

This study had several limitations. We intentionally reduced the number of scenarios to minimize the cognitive burden placed on respondents. Accordingly, it may have been difficult for respondents to make fully informed decisions. Furthermore, we did not collect response integrity data and were therefore unable to analyze characteristics regarding non-respondents.

## Conclusions

Our findings indicate that range of descriptions of lung cancer states can be feasibly evaluated in the South Korean population using either the VAS or SG method. Furthermore, the utility weights generated from this study could be used in economic evaluations of lung cancer interventions for both patients and the general population.

## Additional files

Additional file 1: Lung Cancer Scenarios: Compositions of Health States. (DOCX 18 kb)

Additional file 2: Example: Pulmonary nodule utility study: Interview guide/questionnaire. (DOCX 15 kb)

## Abbreviations

NSCLC: non-small cell lung cancer
SG: Standard gamble
VAS: Visual analog scale
YLD: Years lost to disability
YLL: Years of life lost
