# Supplementary material for: Disease severity-based evaluation of utility weights for lung cancer-related health states in Korea
Source: BMC Cancer. 2018 Nov 8;18:1081. doi: 10.1186/s12885-018-4960-y (PMC6225686; doi:10.1186/s12885-018-4960-y)
Supplement: Supplementary file 1 — Lung Cancer Scenarios: Compositions of Health States. (DOCX 18 kb) [file 12885_2018_4960_MOESM1_ESM.docx]

**Additional file 1**

Lung Cancer Scenarios: Compositions of Health States

1. Lung Cancer (squamous cell carcinoma, 5 Scenarios)
2. Pulmonary Nodule
3. **Stage I Lung Cancer**

**Diagnosis**: You are diagnosed with stage I lung cancer by a doctor after undergoing a chest X-ray, CT scan of the chest, and PET-CT. The cancer is small and has not spread to surrounding tissues.

**Symptoms**: Usually, there are no specific symptoms. You may have a cough and produce phlegm.

**Treatment**: Depending on the results of the pulmonary function test, you may undergo surgery to remove some parts of the lung and surrounding lymph nodes under general anesthesia. Two weeks to 1 month may be needed to recover after surgery. The side effects include bleeding, pain at the surgical site, surgical site infection, and pneumonia. You may be temporarily short of breath after surgery, but appropriate respiratory exercise will gradually relieve this symptom. You may receive chemotherapy to prevent recurrence, if necessary. This will occur for 3–6 months, and you will usually visit an outpatient clinic to receive chemotherapeutic agents. The side effects of chemotherapy include a loss of appetite, nausea, vomiting, hair loss, and infection. After finishing every treatment, you should be examined by a doctor every 6 months, after which the interval between visits will increase gradually.

**Prognosis**: The 5-year survival rate is approximately 60–70%. You may feel anxious about the possibility of cancer recurrence.

1. **Stage II Lung Cancer**

**Diagnosis**: You are diagnosed with stage II lung cancer by a doctor after undergoing a chest X-ray, a CT scan of the chest, bronchoscopy, percutaneous fine needle aspiration, and PET-CT. The cancer is not small, but has not spread to surrounding tissues. Bronchoscopy is usually performed under sedation anesthesia. This procedure is rarely accompanied by complications such as bleeding, pneumothorax, or pneumonia. Percutaneous fine needle aspiration is a diagnostic method that involves inserting a thin needle directly into the mass through the skin. This method also rarely causes complications such as bleeding or pneumothorax.

**Symptoms**: Usually, there are no specific symptoms. You may have a cough and produce phlegm.

**Treatment**: Depending on the results of the pulmonary function test, you may undergo surgery to remove some parts of the lung and surrounding lymph nodes under general anesthesia. Two weeks to 1 month may be required for recovery after surgery. The side effects include bleeding, pain at the surgical site, surgical site infection, and pneumonia. You may be temporarily short of breath after the surgery, but appropriate respiratory exercise will gradually relieve this symptom. You may receive chemotherapy to prevent recurrence, if necessary. This will occur for 3–6 months, and you will usually visit an outpatient clinic to receive the chemotherapeutic agent. The side effects of chemotherapy include the loss of appetite, nausea, vomiting, hair loss, and infection. After finishing every treatment, you should be examined by a doctor every 6 months, after which the interval between visits will increase gradually.

**Prognosis**: The 5-year survival rate is about 40 to 50%. You may feel anxious about the possibility of cancer recurrence.

1. **Stage IIIa Lung Cancer**

**Diagnosis**: You are diagnosed with stage IIIa lung cancer by a doctor after undergoing a chest X-ray, a CT scan of the chest, bronchoscopy, percutaneous fine needle aspiration, and PET-CT. The cancer is not small and has spread to surrounding tissues. Bronchoscopy is usually performed under sedation anesthesia. This procedure is rarely accompanied by complications such as bleeding, pneumothorax, or pneumonia. Percutaneous fine needle aspiration is a diagnostic method that involves inserting a thin needle directly into the mass through the skin. This method also rarely causes complications such as bleeding or pneumothorax.

**Symptoms**: There may be no specific symptoms. You may have a cough and produce phlegm, which may be mixed with blood. Shortness of breath, chest pain, and pneumonia may also occur. You may notice hoarseness in your voice, have difficulty swallowing foods, and observe swelling on the head, neck, shoulder, or arm. You may also experience fatigue, loss of energy, and weight loss.

**Treatment**: Depending on the results of the pulmonary function test, you may undergo surgery to remove some parts of the lung and surrounding lymph nodes under general anesthesia. If necessary, the surgery may be preceded by chemotherapy or radiation therapy to reduce the size of the tumor. Two weeks to 1 month may be needed for recovery after surgery. The side effects include bleeding, pain at the surgical site, surgical site infection, and pneumonia. You may be temporarily short of breath after the surgery, but appropriate respiratory exercise will gradually relieve this symptom.

You will likely receive chemotherapy and radiation therapy to prevent recurrence. The chemotherapy will be administered for 3–4 months, and you will usually visit an outpatient clinic to receive the chemotherapeutic agent. The side effects of chemotherapy include a loss of appetite, nausea, vomiting, hair loss, and infection. Radiation therapy will usually be administered once daily on 5 days per week, for a total of 5–6 weeks. The side effects of radiation therapy include dermatitis, esophagitis (difficulty swallowing and pain during swallowing), radiation pneumonitis, and pulmonary fibrosis. Radiation pneumonitis will normally recover within 4 weeks. However, pulmonary fibrosis can progress gradually, and you may slowly become short of breath. After finishing every treatment, you should be examined by a doctor every 6 months, after which the interval between visits will increase gradually.

**Prognosis**: The 5-year survival rate is approximately 30%. You often feel anxious about the possibility of cancer recurrence.

1. **Stage IIIb Lung Cancer**

**Diagnosis**: You are diagnosed with stage IIIb lung cancer by a doctor after undergoing a chest X-ray, CT scan of the chest, bronchoscopy, percutaneous fine needle aspiration, and PET-CT. The cancer is not small and has spread to surrounding tissues. Bronchoscopy is usually performed under sedation anesthesia. This procedure is rarely accompanied by complications such as bleeding, pneumothorax, or pneumonia. Percutaneous fine needle aspiration is a diagnostic method that involves inserting a thin needle directly into the mass through the skin. This method also rarely causes complications such as bleeding or pneumothorax.

**Symptoms**: There may be no specific symptoms. You may have a cough and produce phlegm, which may be mixed with blood. Shortness of breath, chest pain and pneumonia may also occur. You may notice hoarseness in your voice, have difficulty swallowing foods, and observe swelling on the head, neck, shoulder, or arm. You may also experience fatigue, loss of energy, and weight loss.

**Treatment**: You will receive chemotherapy and radiation therapy. The chemotherapy will occur over 3–4 months, and you will usually visit an outpatient clinic to receive the chemotherapeutic agent. The side effects of chemotherapy include a loss of appetite, nausea, vomiting, hair loss, and infection. Radiation therapy will usually be given once daily on 5 days per week, for a total of 6–7 weeks. The side effects of radiation therapy include dermatitis, esophagitis (difficulty and pain during swallowing), radiation pneumonitis, and pulmonary fibrosis. Radiation pneumonitis will normally recover within 4 weeks. However, pulmonary fibrosis can progress gradually, and you may slowly become short of breath. After finishing every treatment, you should be examined by a doctor every 6 months, after which the interval between visits will increase gradually.

**Prognosis**: The 5-year survival rate is approximately 10–20%. You often feel anxious about the possibility of cancer recurrence.

1. **Stage IV Lung Cancer**

**Diagnosis**: You are diagnosed with stage IV lung cancer by a doctor after undergoing a chest X-ray, CT scan of the chest, bronchoscopy, percutaneous fine needle aspiration, PET-CT, brain MRI, and bone scan. The cancer has spread to the brain and bones. Bronchoscopy is usually performed under sedation anesthesia. This procedure is rarely accompanied by complications such as bleeding, pneumothorax, or pneumonia. Percutaneous fine needle aspiration is a diagnostic method that involves inserting a thin needle directly into the mass through the skin. This method also rarely causes complications such as bleeding or pneumothorax.

**Symptoms**: There may be no specific symptoms. You may have a cough and produce phlegm, which may be mixed with blood. You may also experience shortness of breath, chest pain, and pneumonia. You may notice hoarseness in your voice, have difficulty swallowing foods, and observe swelling on the head, neck, shoulder, or arm. Additionally, you may experience fatigue, loss of energy, and weight loss. You may have pain from the bone metastasis and a headache, nausea, vomiting, and/or paralysis because of the brain metastasis.

**Treatment**: You will receive chemotherapy and radiation therapy. You will usually visit outpatient clinics to receive chemotherapeutic agents. The side effects of chemotherapy include a loss of appetite, nausea, vomiting, hair loss, and infection. Radiation therapy will usually be performed depending on the condition of the patient. The side effects of radiation therapy include dermatitis, esophagitis (difficulty and pain during swallowing), radiation pneumonitis, and pulmonary fibrosis. Radiation pneumonitis will normally recover within 4 weeks. However, pulmonary fibrosis can progress gradually, and you may slowly become short of breath. You will receive additional radiation therapy to your head if a brain metastasis is present. You will also receive radiation therapy to the bone metastases.

**Prognosis**: The 5-year survival rate is <10%. You often feel anxious about the possibility of further cancer metastasis.

1. **Pulmonary nodule**

**Diagnosis**: You are diagnosed with a pulmonary nodule by a doctor after undergoing a chest X-ray, CT scan of the chest, bronchoscopy, and percutaneous fine needle aspiration. Bronchoscopy is usually performed under sedation anesthesia. It is rarely accompanied by complications such as bleeding, pneumothorax, or pneumonia. Percutaneous fine needle aspiration is a diagnostic method involving the insertion of a thin needle directly into the mass through the skin. This method also rarely causes complications such as bleeding or pneumothorax.

**Symptoms**: There may be no specific symptoms.

**Treatment**: CT scans of the chest will be performed at 3–4-month intervals to observe changes in the shape and size of the pulmonary nodule. If the nodule does not change over a set period of time, the interval between imaging studies can be increased. If the pulmonary nodule elicits strong clinical suspicion of a malignant nodule, surgical resection may be performed even if no cancer cells are found.

**Prognosis**: There are no effects on activities of daily living. A pulmonary nodule confers no significant difference in prognosis relative to the normal population. You may feel slightly anxious about the possibility that the nodule may progress to cancer.
