# Supplementary material for: Disease severity-based evaluation of utility weights for lung cancer-related health states in Korea
Source: BMC Cancer. 2018 Nov 8;18:1081. doi: 10.1186/s12885-018-4960-y (PMC6225686; doi:10.1186/s12885-018-4960-y)
Supplement: Supplementary file 2 — Example: Pulmonary nodule utility study: Interview guide/questionnaire. (DOCX 15 kb) [file 12885_2018_4960_MOESM2_ESM.docx]

**Additional file 2**

Example: Pulmonary nodule utility study: Interview guide/questionnaire

VAS: Q1. There is a 100-scaled health status. 100 is the perfect health status without any disease and 0 is the worst health status. Please evaluate the described health status below from 0 to 100.

| **[Description of health state]**  **Pulmonary nodule**  **Diagnosis**: You are diagnosed with a pulmonary nodule by a doctor after undergoing a chest X-ray, CT scan of the chest, bronchoscopy, and percutaneous fine needle aspiration. Bronchoscopy is usually performed under sedation anesthesia. It is rarely accompanied by complications such as bleeding, pneumothorax, or pneumonia. Percutaneous fine needle aspiration is a diagnostic method involving the insertion of a thin needle directly into the mass through the skin. This method also rarely causes complications such as bleeding or pneumothorax.  **Symptoms**: There may be no specific symptoms.  **Treatment**: CT scans of the chest will be performed at 3–4-month intervals to observe changes in the shape and size of the pulmonary nodule. If the nodule does not change over a set period of time, the interval between imaging studies can be increased. If the pulmonary nodule elicits strong clinical suspicion of a malignant nodule, surgical resection may be performed even if no cancer cells are found.  **Prognosis**: There are no effects on activities of daily living. A pulmonary nodule confers no significant difference in prognosis relative to the normal population. You may feel slightly anxious about the possibility that the nodule may progress to cancer. |
| --- |

VAS: Q2. Please compare the health status below with death. In other words, Which is more better health status?

| **□ Death**  **□ Pulmonary nodule**  **Diagnosis**: You are diagnosed with a pulmonary nodule by a doctor after undergoing a chest X-ray, CT scan of the chest, bronchoscopy, and percutaneous fine needle aspiration. Bronchoscopy is usually performed under sedation anesthesia. It is rarely accompanied by complications such as bleeding, pneumothorax, or pneumonia. Percutaneous fine needle aspiration is a diagnostic method involving the insertion of a thin needle directly into the mass through the skin. This method also rarely causes complications such as bleeding or pneumothorax.  **Symptoms**: There may be no specific symptoms.  **Treatment**: CT scans of the chest will be performed at 3–4-month intervals to observe changes in the shape and size of the pulmonary nodule. If the nodule does not change over a set period of time, the interval between imaging studies can be increased. If the pulmonary nodule elicits strong clinical suspicion of a malignant nodule, surgical resection may be performed even if no cancer cells are found.  **Prognosis**: There are no effects on activities of daily living. A pulmonary nodule confers no significant difference in prognosis relative to the normal population. You may feel slightly anxious about the possibility that the nodule may progress to cancer. |
| --- |

SG: Q3. Please compare the following health state with those of being dead. In other words, which of the following two health states do you think is better health?

| **[Description of health state]**  **Pulmonary nodule**  **Diagnosis**: You are diagnosed with a pulmonary nodule by a doctor after undergoing a chest X-ray, CT scan of the chest, bronchoscopy, and percutaneous fine needle aspiration. Bronchoscopy is usually performed under sedation anesthesia. It is rarely accompanied by complications such as bleeding, pneumothorax, or pneumonia. Percutaneous fine needle aspiration is a diagnostic method involving the insertion of a thin needle directly into the mass through the skin. This method also rarely causes complications such as bleeding or pneumothorax.  **Symptoms**: There may be no specific symptoms.  **Treatment**: CT scans of the chest will be performed at 3–4-month intervals to observe changes in the shape and size of the pulmonary nodule. If the nodule does not change over a set period of time, the interval between imaging studies can be increased. If the pulmonary nodule elicits strong clinical suspicion of a malignant nodule, surgical resection may be performed even if no cancer cells are found.  **Prognosis**: There are no effects on activities of daily living. A pulmonary nodule confers no significant difference in prognosis relative to the normal population. You may feel slightly anxious about the possibility that the nodule may progress to cancer. |
| --- |

SG: Q4. (If the participant did not choose a being dead) Imagine living your life with your chosen health state and dying. However, here is a new treatment. When you receive this treatment, you can recover to complete health and live the rest of your life, but this treatment has side effects and may die soon after treatment. If the possibilities are as follows: Would you like to receive this treatment? Do not you accept it? Or do you think the two choices are the same?

| **[Description of health state]**  **Pulmonary nodule**  **Diagnosis**: You are diagnosed with a pulmonary nodule by a doctor after undergoing a chest X-ray, CT scan of the chest, bronchoscopy, and percutaneous fine needle aspiration. Bronchoscopy is usually performed under sedation anesthesia. It is rarely accompanied by complications such as bleeding, pneumothorax, or pneumonia. Percutaneous fine needle aspiration is a diagnostic method involving the insertion of a thin needle directly into the mass through the skin. This method also rarely causes complications such as bleeding or pneumothorax.  **Symptoms**: There may be no specific symptoms.  **Treatment**: CT scans of the chest will be performed at 3–4-month intervals to observe changes in the shape and size of the pulmonary nodule. If the nodule does not change over a set period of time, the interval between imaging studies can be increased. If the pulmonary nodule elicits strong clinical suspicion of a malignant nodule, surgical resection may be performed even if no cancer cells are found.  **Prognosis**: There are no effects on activities of daily living. A pulmonary nodule confers no significant difference in prognosis relative to the normal population. You may feel slightly anxious about the possibility that the nodule may progress to cancer. |
| --- |
